# A unique tolerizing dendritic cell phenotype induced by the synthetic triterpenoid CDDO-DFPA (RTA-408) is protective against EAE

**DOI:** 10.1038/s41598-017-06907-4

**Published:** 2017-08-29

**Authors:** Hsi-Ju Wei, Tej K. Pareek, Qi Liu, John J. Letterio

**Affiliations:** 1 Department of Biochemistry, School of Medicine, Case Western Reserve University, Cleveland, OH 44106 USA; 2 Department of Pediatrics, Division of Pediatric Hematology/Oncology, Case Western Reserve University, Cleveland, OH 44106 USA; 3 Angie Fowler Cancer Institute, Rainbow Babies & Children’s Hospital, University Hospitals, Cleveland, OH 44106 USA

## Abstract

Tolerogenic dendritic cells (DCs) have emerged as relevant clinical targets for the treatment of multiple sclerosis and other autoimmune disorders. However, the pathways essential for conferring the tolerizing DC phenotype and optimal methods for their induction remain an intense area of research. Triterpenoids are a class of small molecules with potent immunomodulatory activity linked to activation of Nrf2 target genes, and can also suppress the manifestations of experimental autoimmune encephalomyelitis (EAE). Here we demonstrate that DCs are a principal target of the immune modulating activity of triterpenoids in the context of EAE. Exposure of DCs to the new class of triterpenoid CDDO-DFPA (RTA-408) results in the induction of HO-1, TGF-β, and IL-10, as well as the repression of NF-κB, EDN-1 and pro-inflammatory cytokines IL-6, IL-12, and TNFα. CDDO-DFPA exposed DCs retained expression of surface ligands and capacity for antigen uptake but were impaired to induce Th1 and Th17 cells. TGF-β was identified as the factor mediating suppression of T cell proliferation by CDDO-DFPA pretreated DCs, which failed to passively induce EAE. These findings demonstrate the potential therapeutic utility of CDDO-DFPA in the treatment and prevention of autoimmune disorders, and its capacity to induce tolerance via modulation of the DC phenotype.

## Introduction

Antigen-presenting cells (APCs) or dendritic cells (DCs) are central players in the development and maintenance of immunity and tolerance^1–3^. Efforts to exploit their potential as cellular therapies range from the induction of tumor immunity to the establishment of transplant tolerance and the suppression of autoimmunity^4–6^. Successful pursuit of these applications requires fully understanding the factors influencing DC maturation and function^7–10^, as well as the soluble factors that mediate their effects on T cells and other immune cells^11^. Agents that repress DC costimulatory molecule expression confer a tolerogenic DC phenotype^12, 13^. Further, there is increasing appreciation of the importance of intracellular enzymes such as heme oxygenase-1 (HO-1) and soluble, secreted factors that range from the HO-1 enzymatic reaction product carbon monoxide (CO)^14–16^, to suppressive cytokines such as transforming growth factor-beta (TGF-β)^17^, IL-10, and other modulators of vascular and lymphocyte function, such as endothelin-1 (EDN-1)^18^.

Triterpenoids are a broad class of small molecules that include ursolic acid, oleanolic acid, celastrol, and others with pentacyclic motif and potent immune modulating activity^19–21^. Synthetic derivatives of natural triterpenoids have been developed and extensively studied for their potential in cancer chemoprevention^22^. Their efficacy as chemopreventives in numerous preclinical models of carcinogenesis has been directly linked to their capacity to modulate the expression of antioxidant and stress response proteins whose expression is regulated by the transcription factor nuclear factor (erythroid-derived 2)-like 2 (Nrf2)^23, 24^. However, the suppression of carcinogenesis has also been linked to inhibition of pro-inflammatory mediators such as nuclear factor kappa B (NF-κB) and Stat3^25^, to the induction of tumor suppressor pathways regulated by the prostaglandin degrading enzyme 15-hydroxyprostaglandin dehydrogenase (15-PGDH) and by TGF-β^26^, and through potent transcriptional repression of inducible nitric oxide synthase (iNOS)^27^.

These activities predict the potential utility of triterpenoids in the treatment and prevention of autoimmune and inflammatory disorders. Studies by our laboratory and by many others have shown triterpenoid efficacy in the prevention of lethality in preclinical models of sepsis and graft versus host disease^28–31^, and in the reversal of manifestations of neuroinflammation in models of neurodegenerative diseases, including EAE^32^. We have shown suppression of EAE by synthetic triterpenoids is linked to inhibition of Th1 and Th17 mRNA and cytokine production and to the capacity of triterpenoids to promote myelin repair^32^. However, the effects of triterpenoids on DC function in this context have not been carefully explored.

We hypothesized that triterpenoids suppress autoimmune and alloreactive T cell responses through direct effects on DC function. We show the synthetic triterpenoid 2-cyano-3,12-dioxooleana-1,9-dien-28-oic acid-difluoro-propyl-amide, (CDDO-DFPA, RTA-408) induced a profile of DC gene expression characterized by the induction of mediators of a tolerogenic phenotype including HO-1, TGF-β and IL-10, without altering DC antigen uptake or expression of cell surface costimulatory molecules. Importantly, *ex vivo* expanded, CDDO-DFPA exposed DCs failed to passively induce EAE, suggesting the induction of a unique tolerogenic DC (TolDC) phenotype. The data presented here suggest CDDO-DFPA and related triterpenoids may prove useful for induction of TolDCs, including the *ex-vivo* expansion of autologous TolDCs for therapeutic application.

## Results

### CDDO-DFPA suppresses development of EAE

**Figure 1:**
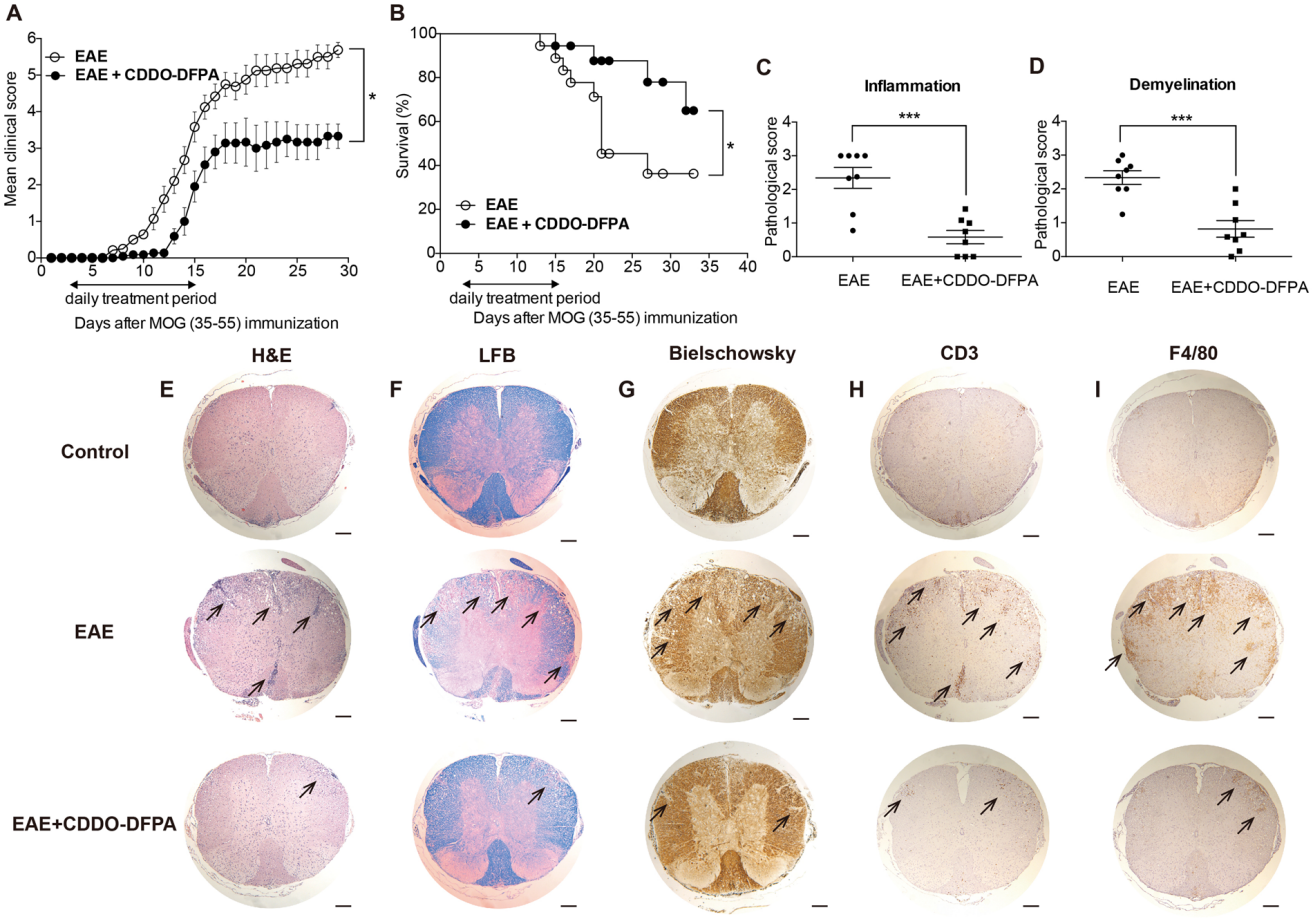
CDDO-DFPA is protective against clinical pathology of EAE. EAE was induced in age-matched female C57BL/6 mice (8 to 10 weeks old), by MOG (35–55) immunization. Pertussis toxin ﻿(PTX) was also injected immediately and again 2 days later. CDDO-DFPA was administered (i.p. injection) daily from day 3 to day 15. (**A**) A clinical score was assigned to each mouse daily. All data are presented as the mean ± S.E.M. *P < 0.05. Multiple t-tests with Holm-Sidak analysis. (**B**) Survival curve for immunized mice (Kaplan-Meier survival curve followed by the Mental-Cox log-rank test within 35 days (n = 11–12 mice in each group). *P < 0.05. Mice were sacrificed at day 21 and representative sections of lumbar spinal cord were prepared from control mice and from mice immunized with MOG and subsequently treated with either CDDO-DFPA or vehicle control. Tissues were stained with hematoxylin and eosin (H&E) to assess inflammation (**C**,**E**), with Luxol fast blue (LFB) to assess myelin content (**D**,**F**), and with Bielschowsky stain to measure axonal loss (**G**). CD3 and F4/80 antibodies were used for immunohistochemical localization of T cells (**H**) and macrophages (**I**), respectively (each indicated by arrows). A pathologist blinded to subject identity scored sections taken from each animal for H&E inflammation (**C**) and LFB demyelination (**D**) on the scale of 0 to 3. Scale bars, 100 μm. Quantification data in panel C and D were presented as the mean ± S.E.M. (n = 8 mice in each group). ***P < 0.001, Unpaired student t-test.

We previously reported the therapeutic utility of various derivatives of the synthetic triterpenoid CDDO in EAE^32^. Here we examine the potential of the more recently developed CDDO derivative CDDO-DFPA, and the relevance of timing of exposure relative to MOG (35–55) immunization and T cell priming. Manifestations of EAE typically appear by day 7 following immunization and activated T cells, and DCs have each been detected infiltrating the central nervous system (CNS) at day 5^33^. Therefore, we began daily intraperitoneal (i.p.) administration of CDDO-DFPA at day 3, extending treatment through day 15. The data show that limited administration of CDDO-DFPA during this T cell priming phase significantly delayed disease onset and reduced the severity of EAE when compared to the control group (Fig. 1A), and significantly improved overall survival in mice with EAE from 38% to 75% (Fig. 1B). Since Nrf2 is a known target of CDDO derivatives^23, 24^ and also known to play a significant role during EAE^34^, we next tested the effect of CDDO-DFPA treatment on the induction and progression of EAE in Nrf2 knockout mice. As expected, following MOG immunization, we observed exacerbated clinical signs and reduced survival of Nrf2 knockout mice compared to their wild type littermate controls. However; regardless of their genotypic differences CDDO-DFPA treatment was equivalently able to improve survival and reduce clinical manifestations of the disease in both Nrf2 knockout and wild type mice (Figure S1). These results demonstrate that observed protective effect of CDDO-DFPA in EAE mice is principally Nrf2 independent. CDDO-DFPA treated spinal cords exhibited reduced inflammation (Fig. 1C,E), demyelination (Fig. 1D,F) and axon damage (Fig. 1G). It is well documented that during the initial phase of MS and EAE, peripheral macrophages infiltrate and accumulate in the CNS, where, together with residential microglia, they participate in the induction and development of disease^35^. These polarized macrophages also potent modulators of T cell priming and of T cell recruitment into the CNS. In order to assess the potential role of CDDO-DFPA in immune modulation during EAE we performed immunohistochemical analyses with anti-CD3 (for T cells) and anti-F4/80 (for macrophages and microglia) antibodies on adjacent, serial lumbar spinal cord sections. The results show a significant reduction in the number of infiltrating T cells (Fig. 1H) and macrophages (Fig. 1I), in CDDO-DFPA treated mice when compared to their respective controls. These results suggest that CDDO-DFPA treatment influences the immune microenvironment to suppress the course of disease.

### CDDO-DFPA suppresses NF-κB signaling in BMDCs

**Figure 2:**
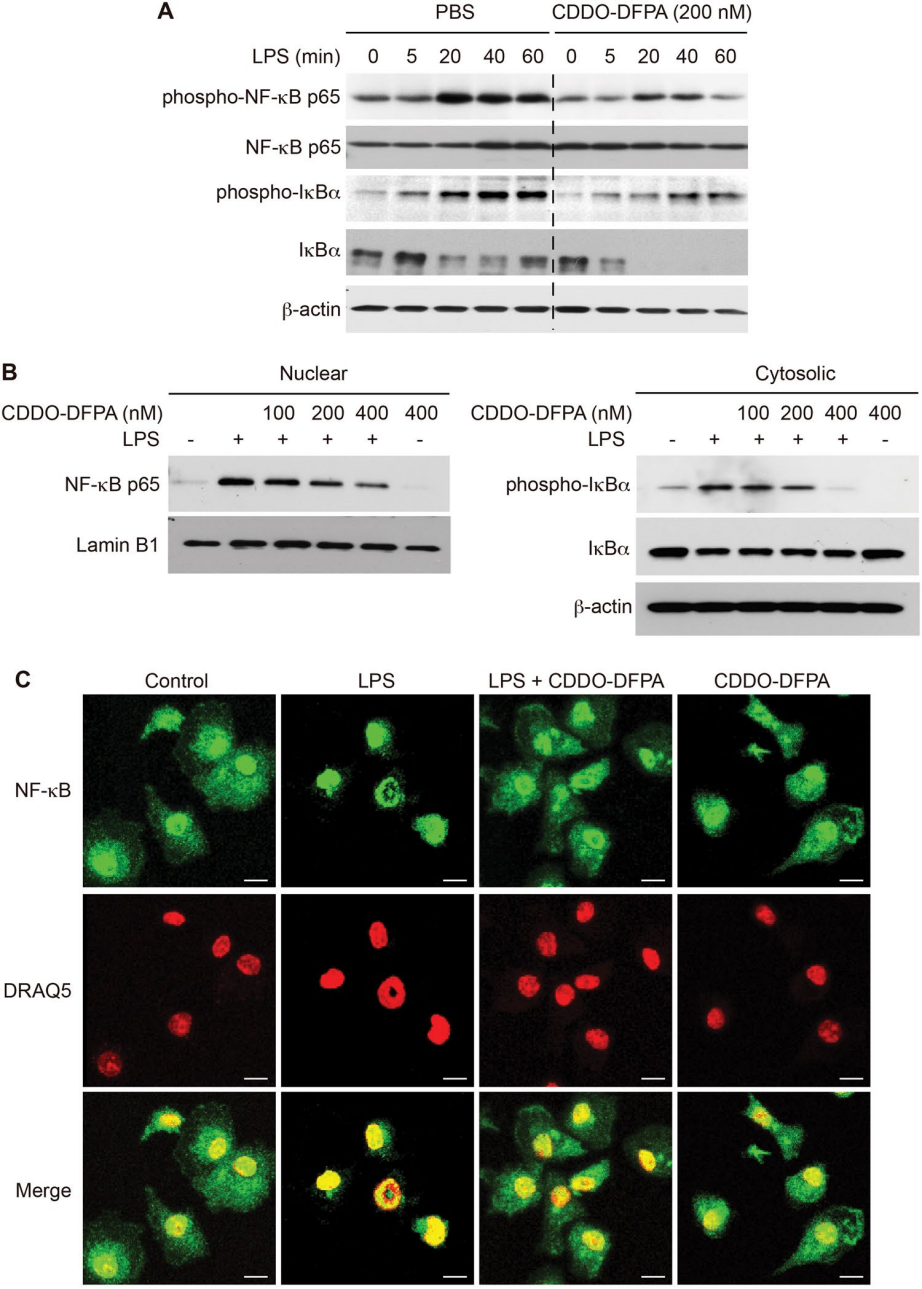
CDDO-DFPA suppresses NF-κB signaling in LPS-activated BMDCs. (**A**) CDDO-DFPA impairs time-dependent induction of NF-κB in BMDCs expanded from the normal bone marrow of C57BL/6 mice that were pretreated in either the presence or absence of CDDO-DFPA (200 nM) for 1 hour before LPS (100 ng/ml) treatment for the indicated intervals. (**B**) BMDCs were pretreated in the presence or absence of CDDO-DFPA (100–400 nM) for 1 hour, followed by stimulation with LPS for 10 mins (100 ng/ml). The cytosolic, nuclear, and total cellular lysates were analyzed for phospho- and total NF-κB p65 and IκBα, β-actin, and Lamin B1 expression by Western blotting. All depicted blots are cropped and respective full-length blots are presented in the Supplementary Figure S6. (**C**) BMDCs were pretreated in the presence or absence of CDDO-DFPA (400 nM) for 1 hour, followed by stimulation with LPS for 10 mins (100 ng/ml). Cells were fixed and stained with NF-κB and DRAQ5 (nucleus), and images were acquired by confocal microscopy. All experiments were repeated a minimum of three times.

DC activation is required for the initiation of EAE^36^. Therefore, we next examined the effect of CDDO-DFPA on DC activation *in vitro*, initially focusing on the regulation of signaling via the transcription factor NF-κB, which is considered essential for DC activation and function^37^. NF-κB deficient mice show resistance to EAE induction, due to the failure of MOG-specific T cells to differentiate into either Th1 or Th2 cells in these mice^38^. We isolated and pretreated BMDCs in either the presence or absence of 200 nM CDDO-DFPA for 1 hour before stimulation with 100 ng/ml of LPS (Fig. 2A). LPS exposure requires the transcription factor NF-κB to fully activate DCs^39^. In the resting state, NF-κB is sequestered and bound with IκBα in the cytoplasm. During LPS stimulation, both NF-κB and IκBα are rapidly phosphorylated. IκBα disassociates from NF-κB and is degraded, allowing NF-κB to translocate to the nucleus^40^. As shown in Fig. 2A, LPS treatment induced phosphorylation of NF-κB and IκBα in BMDCs after 20 mins and 5 mins, respectively (left panel). Although the phosphorylation pattern was unaltered, the overall phosphorylation of NF-κB and IκBα was reduced upon CDDO-DFPA treatment during the observed time course, compared to their respective controls (right panel). We observed increased IκBα degradation upon CDDO-DFPA treatment (Fig. 2A), which may be due to a negative feedback for NF-κB driving IκBα expression, as previously demonstrated for CDDO-DFPA and for the approved MS therapeutic, dimethyl fumarate^22, 41^. When BMDCs were pretreated with or without CDDO-DFPA (100 to 400 nM) for 1 hour before stimulation with 100 ng/ml of LPS for 10 mins (Fig. 2B), CDDO-DFPA reduced nuclear translocation of NF-κB and the cytoplasmic level of phospho-IκBα. Immunocytochemical analysis (Fig. 2C) showed rapid translocation of NF-κB to the nucleus within 10 mins of LPS treatment. However, brief exposure to CDDO-DFPA prior to stimulation with LPS led to retention of NF-κB in the cytoplasm. These results suggest CDDO-DFPA regulates DC activation through modulation of NF-κB signaling.

### A unique CDDO-DFPA-induced cytokine signature in LPS-activated BMDCs

**Figure 3:**
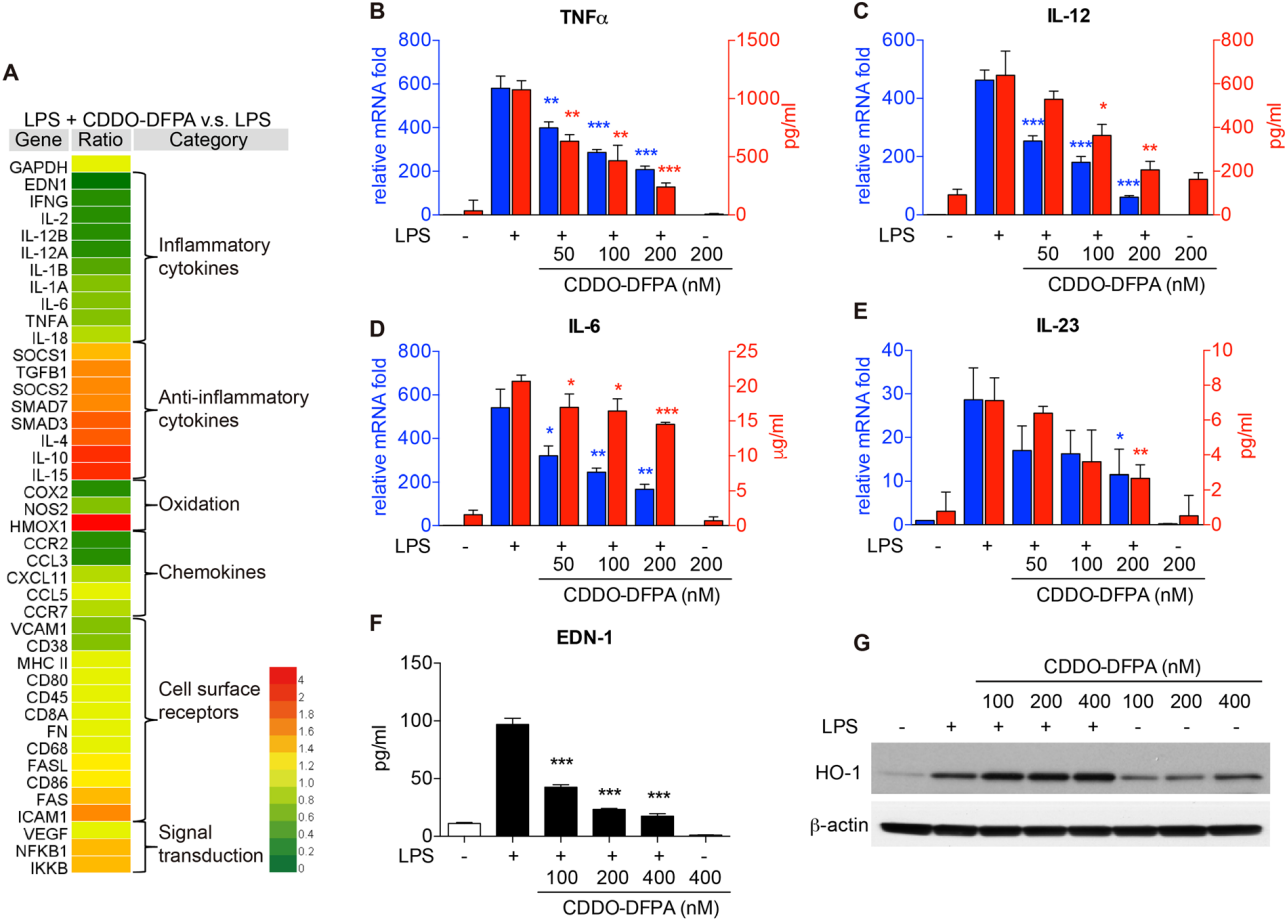
A unique CDDO-DFPA-induced transcriptome in LPS-activated BMDCs. (**A**) Cells were pretreated in the presence or absence of CDDO-DFPA (200 nM) for 1 hour before exposure to LPS (100 ng/ml for 3 hours). Cells were harvested, and RNA was extracted for qRT-PCR array. Expression of each gene was normalized by the control gene (GAPDH) in its own group and then normalized to the average value of each gene within groups. A total of 41 genes known to be related to DC maturation and mediators of DC modulated T cell responses were sorted and classified from 69 genes. The heat map was drawn using the HemI (Heat map illustrator) with the default value. (**B**–**E**) Cells were pretreated in the presence or absence of CDDO-DFPA (50–200 nM) for 1 hour prior to addition of LPS (100 ng/ml), and either harvested for RNA extraction (4 hours) or allowed to condition culture medium for 24 hours prior to collection for cytokine analyses. The levels of TNFα, IL-12, IL-6, and IL-23 were measured by qRT-PCR and ELISA. (**F**,**G**) Conditioned medium and cell protein lysate (12 hours) were collected for analyses. The levels of EDN-1, HO-1, and β-actin expression were analyzed by ELISA and Western blotting. Depicted blots are cropped and respective full-length blots are presented in the supplementary Figure S6. The results are expressed as mean ± S.D. of three experiments. *P < 0.05, **P < 0.01, ***P < 0.001 compared with the LPS-treated groups. Unpaired student t-test.

To define the impact of CDDO-DFPA on the expression of NF-κB target genes, and on the expression of factors that may influence DC viability and DC-T cell interactions, we assessed an immune response gene profile using quantitative real-time PCR (qRT-PCR) arrays. As depicted in in Figure S2, CDDO-DFPA induced alterations in the expression of 69 different genes when compared to the control group. In the cells exposed to CDDO-DFPA alone, we observed a robust induction of the *HMOX1* gene; a predicted effect of CDDO-DFPA as *HMOX1* is regulated by Nrf2^22^. Other genes in the group exposed to CDDO-DFPA alone were either unchanged or down-regulated. Among these genes, 41 have been related to DC maturation or recognized as effectors of DC modulation of the T cell response. These genes are sorted and classified in groups of inflammatory cytokines, anti-inflammatory cytokines, oxidation, chemokines, cell surface receptors, and signal transduction (Fig. 3A). A comparison of BMDCs exposed to CDDO-DFPA plus LPS to those exposed to LPS alone in this gene expression array showed that CDDO-DFPA treatment significantly reduced the BMDC expression of pro-inflammatory cytokine genes such as *IFN-γ*, *IL-12*, *EDN1, IL-18, IL-2, TNFα, IL-1, IL-6*, and *IL-23* in LPS-activated BMDCs. Both *IFN-γ* and *IL-12* are necessary for Th1 T cell differentiation the latter three (*IL-1*, *IL-6, and IL-23*) are necessary for Th17 cell differentiation, and together these cytokines are the essential mediators of the inflammatory process in both EAE and MS^42, 43^. BMDCs treated with CDDO-DFPA also had increased expression of anti-inflammatory cytokine genes such as *IL-4, SOCS, SMAD3, IL-15, IL-10*, and *TGF-β*. *IL-4* promotes the differentiation of CD4 T cells toward the Th2 phenotype, and *IL-10* and *TGF-β* are known to exert anti-inflammatory activity and to suppress autoimmunity through mechanisms that include the induction of Treg^44^. CDDO-DFPA also decreased the expression of the pro-inflammatory genes (*COX2*, *NOS2*), and of several chemokine genes. However, genes encoding cell surface receptors and costimulatory ligands remained mostly unchanged after CDDO-DFPA treatment. Importantly, the observed CDDO-DFPA repression of inflammatory cytokines for Th1 and Th17 differentiation was confirmed by both qRT-PCR and by ELISA (Fig. 3B–E). CDDO-DFPA also reduced EDN-1 production and increased HO-1 expression in BMDCs as observed by ELISA and Western blotting (Fig. 3F,G). It is noteworthy that the distinctive IL-12^−^ IL-10^+^ cytokine production profile^45^, the inhibition of EDN-1^46^, and induction of HO-1 expression^16^ induced by CDDO-DFPA are all known to endow DCs with tolerogenic function. Surprisingly, while TolDCs typically express low levels of surface costimulatory ligands, we found CDDO-DFPA neither suppressed LPS-induced surface ligand expression, including MHC II, CD80, CD86, and PD-L1 (Figure S3), nor the capacity of BMDCs for antigen uptake (Figure S4). These results suggest the potential for a unique DC-dependent mechanism through which CDDO-DFPA modulates T cell proliferation and differentiation.

### CDDO-DFPA induced TGF-β mediates BMDC suppression of T cell proliferation

**Figure 4:**
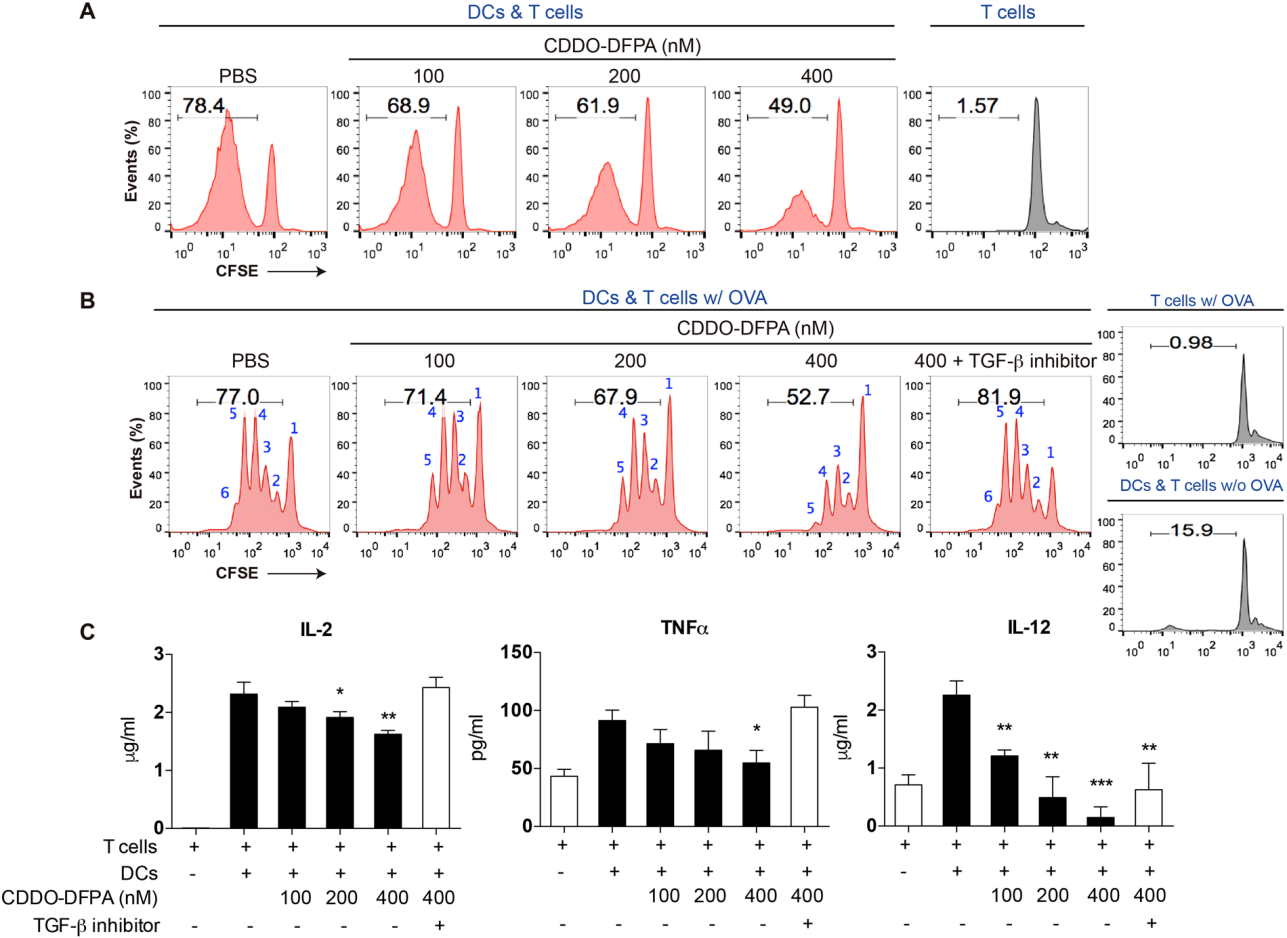
CDDO-DFPA exposed DCs suppress T cell proliferation. DCs were pretreated with CDDO-DFPA (400 nM) for 1 hour only, then washed and co-cultured with CFSE stained T cells at a 1:10 ratio. (**A**) In an *in vitro* model of allogeneic T cell stimulation, splenic T cells and DCs were isolated from C57BL/6 and BALB/c mice respectively and T cell proliferation was determined by flow cytometry at day 6. (**B**) In a syngeneic model, splenic T cells and DCs were isolated from C57BL/6 OTII transgenic mice and C57BL/6 mice respectively. CDDO-DFPA pretreated DCs were co-cultured with CFSE stained T cells with (w/) or without (w/o) OVA addition during incubation in presence or absence of TGF-β receptor inhibitor EW-7197 (5 μM). T cell proliferation was determined by flow cytometry at day 2. Graphs depict the percentage of dividing T cells relative to numbers T cell division. (**C**) Medium conditioned by the co-culture DCs and C57BL/6 OTII transgenic T cells was also collected to assay levels of IL-2, TNFα, and IL-12 by ELISA. The data is a representation of 3 independent experiments. The results in panel C are expressed as mean ± S.D. *P < 0.05, **P < 0.01, ***P < 0.001 compared with the co-cultured untreated group. Unpaired student t-test.

DCs promote T cell differentiation and proliferation through their engagement of costimulatory ligands and through the elaboration of cytokines and other soluble mediators^47^. Our data thus far suggest that CDDO-DFPA has the capacity to modulate the T cell response by altering gene expression and function of DCs. Therefore, we examined how CDDO-DFPA modified DC-mediated T cell proliferation, using *in vitro* models of both allogeneic and syngeneic stimulation. Isolated DCs were pretreated with CDDO-DFPA and washed prior to co-culture with CFSE stained T cells, either with OVA peptide (syngenic) or without (allogenic). CDDO-DFPA treatment of DCs significantly reduced the percentage of proliferated T cells in both models (Fig. 4). Furthermore, T cells incubated with CDDO-DFPA prior to co-culture with DCs retained their full proliferative capacity (Figure S5). These results (Figs 4 and S3–S5) suggests that the observed reduction in T cell proliferation in cultures with DCs exposed to CDDO-DFPA is due to altered cytokine secretion by DCs, which can be a critical determinant of T cell proliferation^48^. Moreover, we found this reduction in proliferation of T cells incubated with CDDO-DPFA exposed DCs is mediated in part by TGF-β as the addition of the specific inhibitor of the TGF-β type I receptor kinase, EW-7197, partially reversed inhibition of T cell proliferation by conditioned medium from DCs cultured in the presence of CDDO-DFPA (Fig. 4B). This response was accompanied by complete restoration of IL-2 and TNFα and partial recovery of IL-12 cytokine production (Fig. 4C) as evident by cytokine ELISA performed on these co-cultured supernatants. These results are consistent with the known ability of TGF-β to suppress IL-2^49^, TNFα^50^, and IL-12^51^ expression.

### CDDO-DFPA impaired DC induction of Th17 T cells but not of Treg cells *in vitro*

**Figure 5:**
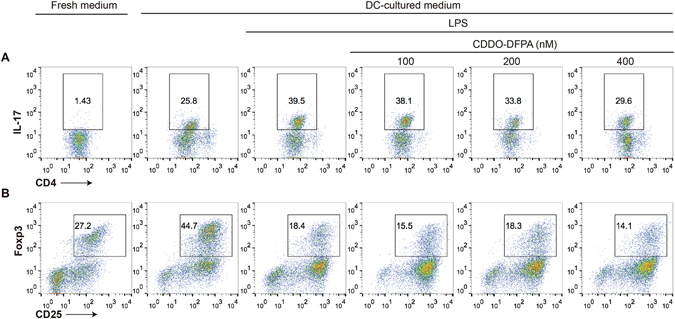
CDDO-DFPA-exposed DCs suppress Th17 induction via soluble mediators. BMDCs were pretreated in the presence or absence of CDDO-DFPA (100–400 nM) for 1 hour and washed before incubation with LPS (100 ng/ml) for 24 hours. DC conditioned medium was collected and stored in −80 °C. Splenic CD4+ T cells were isolated from C57BL/6 mice and incubated under the indicated conditions with the addition of DC-cultured medium. T cells were stimulated for 2 days (Th17) or 3 days (Treg) with plate-bound CD3 and CD28 antibodies and in the presence of differentiation factors (TGF-β and IL-6 for Th17, TGF-β only for Treg). Subsets of IL-17+ Th17 (**A**) and CD25+/Foxp3+ Treg (**B**) were analyzed by flow cytometry. Similar results were obtained in three independent experiments.

Th17 cells, characterized by IL-17 cytokine production, promote a potent pro-inflammatory role in the pathogenesis of MS^52^. Regulatory T cells (Treg) are a specific suppressor subset of CD4+ T cells characterized by expression of the IL-2 receptor α-chain (CD25) on their cell surface, and by nuclear expression of the transcription factor, forkhead box p3 (﻿Foxp3)^53^. An increased frequency of Th17 cells and reduced frequency of Treg T cells in the peripheral blood is known to correlate with disease severity in MS patients^54^. To assess how CDDO-DFPA exposed BMDCs influence T cell differentiation, naïve T cells were activated by CD3/28-stimulation in the culture medium collected from BMDCs alone (control) or BMDCs treated with or without LPS in the presence or absence of CDDO-DFPA (Fig. 5). The medium from LPS-stimulated BMDCs significantly promoted Th17 differentiation (39.5%) when compared to the control medium (25.8%). However, medium from LPS-stimulated BMDCs exposed to CDDO-DFPA (100–400 nM) suppressed Th17 T cell differentiation in a dose-dependent manner (38.1% to 29.6%) (Fig. 5A). In contrast, the repression of Treg development observed in cultures with medium from LPS-stimulated BMDCs (from 44.7% to 18.4%) was not influenced by the exposure of BMDC to CDDO-DFPA (Fig. 5B). Despite the observed increase in TGF-β release, we failed to observe any significant impact of CDDO-DFPA treatment on the induction of Treg. This result can readily be explained by the abundance of IL-6 present in these cultures, which is at a level sufficient to suppress TGF-β-mediated Treg induction. As shown in Fig. 3, we observed more than 20 µg/ml of IL-6 in supernatants of LPS treated BMDCs, and this level reduced to only 15 µg/ml in the presence of CDDO-DFPA. It is known that IL-6 cooperates with TGF-β to induce development of Th17 cells from naıve T cells. However, in contrast, IL-6 inhibits TGF-β induced Treg differentiation through mechanisms that include Stat3 repression of Smad3-dependent TGF-β induction of Foxp3^55^. Our data indicates that CDDO-DFPA can significantly impaired the capacity of LPS-activated DCs to induce a Th17 response, but had no effect on BMDC-induced Treg differentiation in this assay due to the abundance of IL-6.

### Administration of CDDO-DFPA alters T cell differentiation during EAE induction

**Figure 6:**
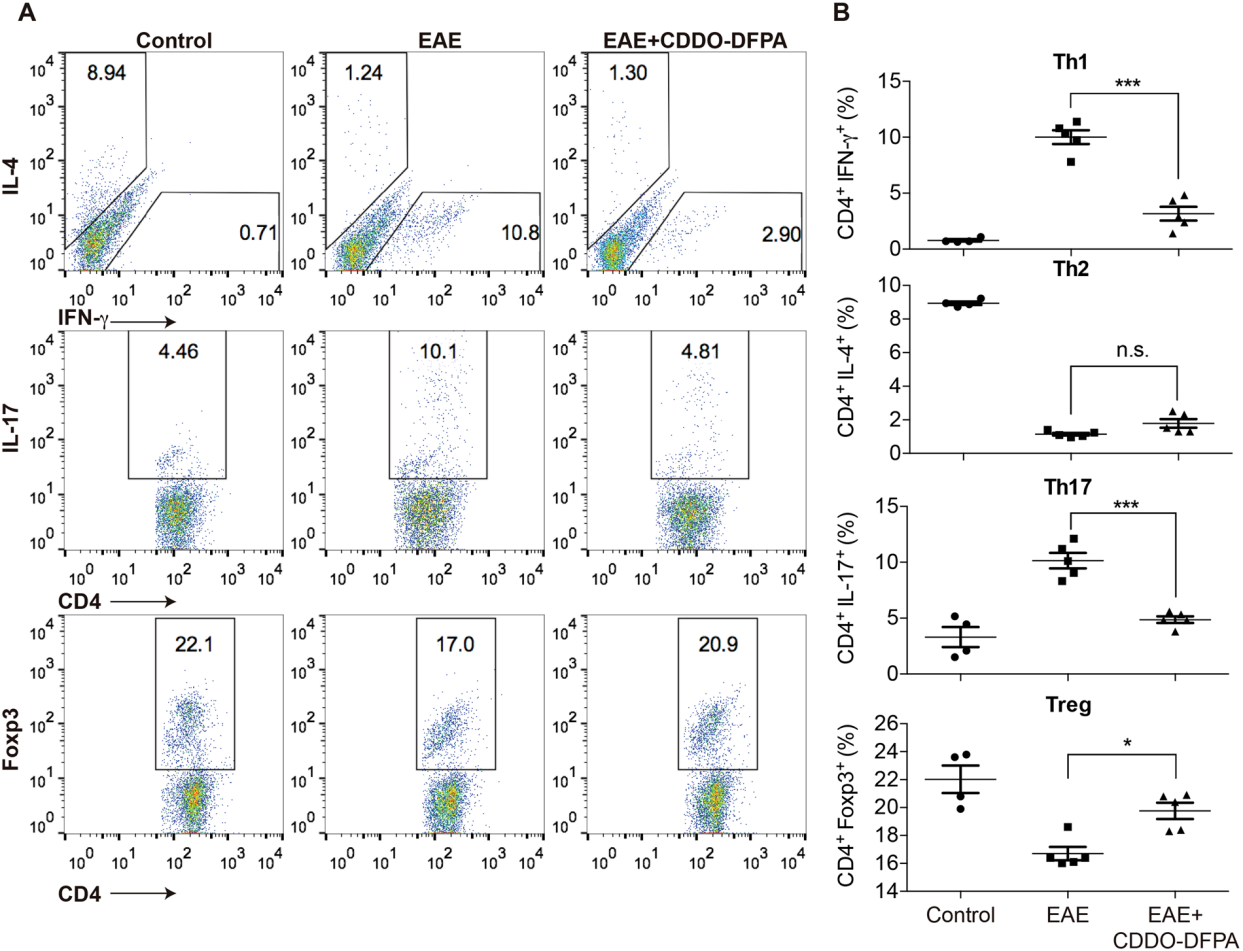
CDDO-DFPA increased Treg and suppressed Th1 and Th17 responses during EAE induction. (**A**) Mice were immunized with MOG (35–55) and treated with either vehicle control or CDDO-DFPA daily from day 3 to day 15 by i.p injection. Mice were sacrificed at day 17 and splenocytes were harvested and stimulated with PMA/ionomycin/Golgistop for 4 hours and subjected to flow cytometry to determine the frequency of Th1, Th2, Th17, and Treg subsets among CD4+ T cells based on their expression of IFN-γ, IL-4, IL-17, and Foxp3, respectively. (**B**) Quantification of data was presented as the mean ± S.E.M. (n = 4–5 mice in each group). *P < 0.05, ***P < 0.001, One-way ANOVA with the Bonferroni corrections.

Th1 and Th17 cells are considered the two pathogenic T cell subsets in MS^56^, and both contribute to the induction of EAE in mice, albeit through different mechanisms^57^. These effector T cell subsets are both regulated by Treg in EAE^58^. Therefore, we assessed the impact of the exposure to CDDO-DFPA in *in vivo* T cell differentiation following MOG immunization, following the same protocol as in Fig. 1. As shown in Fig. 6, mice treated with CDDO-DFPA-during the induction phase of EAE showed a significantly lower percentage of Th1 and Th17 T cell differentiation and a higher proportion of Treg cells when compared to control mice, but no significant difference in Th2 populations. These results are consistent with our data showing DCs exposed to CDDO-DFPA were less able to promote Th17 differentiation *in vitro*.

### CDDO-DFPA exposed DCs pulsed with MOG (35–55) fail to passively induce EAE

**Figure 7:**
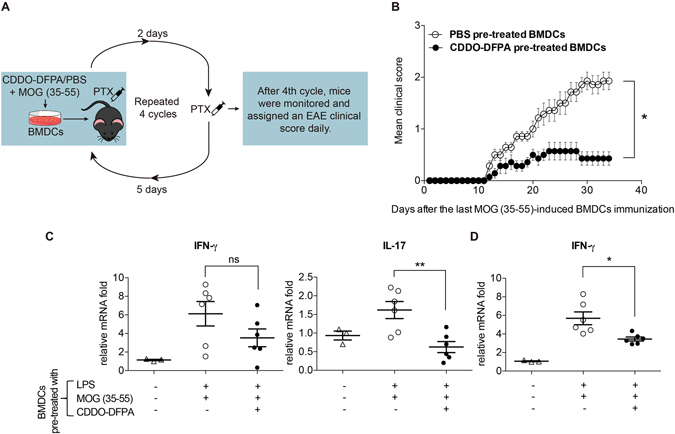
Passively DC-induced EAE is abrogated by DC exposure to CDDO-DFPA. (**A**) EAE induction by MOG (35–55)-pulsed BMDCs. Mature BMDCs were treated in the presence or absence of CDDO-DFPA (400 nM) and subsequently pulsed with MOG (35–55) for 4 hours. A total of 200 μl of 2 × 10^6^ cells were administered by subcutaneous injected into the flank region of C57BL/6 mice once each week for a total of four injections. Each time, PTX was administered by i.p. injection immediately and again 2 days later (day 0 on 4th cycle). (**B**) Clinical scores were recorded after all four injections, using standard criteria. All data were presented as the mean ± S.E.M. *P < 0.05. Multiple t-tests with Holm-Sidak analysis (n = 7 mice in each group). (**C** and **D**) These mice were sacrificed on day 17 and mRNA expression of IFN-γ and IL-17 in the spleen (**C**) and IFN-γ mRNA expression in the lumbar spinal cord were measured by qRT-PCR. The results in panel C and D were presented as the mean ± S.E.M. (n = 3–6 mice in each group). *P < 0.05, **P < 0.01, One-way ANOVA with the Bonferroni corrections.

Although CDDO-DFPA did not repress DC expression of costimulatory molecules, it did induce a profile of soluble mediators that act to repress Th1 and Th17 differentiation, suppress T-cell proliferation and thus may confer a tolerogenic capacity to DCs. To further explore this potential, we examined the capacity of CDDO-DFPA treated DC to passively induce EAE. In this model, EAE is induced by repeated injections of *ex vivo* expanded, LPS activated CD11c+ DC pulsed with MOG (35–55) (Fig. 7A). Clinical signs of EAE were assessed daily as described in the method section and control groups began to exhibit clinical symptoms by week 4 (Fig. 7B). However, in the group receiving DCs exposed to CDDO-DFPA before LPS stimulation showed a significant reduction in the severity of clinical symptoms and a delay in the onset of disease when compared to the control group, which received BMDCs that were pretreated with PBS only. Furthermore, we observed reduced IFN-γ levels in spleen and lumbar spinal cord and significantly suppressed IL-17 in spleen of mice receiving DCs exposed to CDDO-DFPA before LPS stimulation (Fig. 7C,D). These results confirm that CDDO-DFPA has direct impact on DCs which contributes to the reduced clinical signs of EAE.

## Conclusion

In summary, our data suggest that the exposure of DCs to CDDO-DFPA confers a phenotype that includes a diminished capacity to promote pro-inflammatory T cell differentiation *in vitro* and *in vivo*, and an attendant inability to optimally elicit an antigen-specific T cell response to MOG *in vivo*. These observations establish the therapeutic potential of CDDO-DFPA and other related triterpenoids for tolerance induction in DCs, including the *ex-vivo* expansion of autologous TolDCs to manage autoimmune and other inflammatory disorders.

## Discussion

The present study provides evidence that DCs are an important cellular mediator of the immune modulating properties of small molecules in the triterpenoid family. Moreover, the data reveal the capacity of triterpenoids to confer a unique DC phenotype that may be tolerogenic, as demonstrated in the context of EAE.

The data presented for CDDO-DFPA extend our previous studies with this class of synthetic triterpenoids and highlight the therapeutic potential of this derivative, which has already been well characterized for its antioxidant^59^ and anti-inflammatory activities^22^, and has entered early phase clinical trials^60^. Other CDDO derivatives have been shown to inhibit the NF-κB pathway through direct binding to IKKβ^61–63^, but our analyses with CDDO-DFPA suggest a particular relevance for this effect in DCs. The concentration of CDDO-DFPA used in this study (0.05–0.4 μM) is well below the IC50 values (0.8–1.4 μM) for DCs as determined by cell viability assays (Figure S6). Consistent with our results, others have shown significant inhibition of NF-κB activity by CDDO-DFPA in the dose range of 0.25–2 μM^22^, suggesting that CDDO-DFPA may be a more potent immune modulator than other CDDO derivatives^62, 63^.

While NF-κB activity may be important in regulating the switch between the tolerogenic and immune activating potential of DCs, it may not serve as the primary mediator of the triterpenoid response. For example, the abundant HO-1 expression is a hallmark of immature dendritic cells and has been shown to play a major role in mediating the induction of T cell tolerance by DCs^15, 16^, and Nrf-2-dependent transcriptional induction of HO-1 by triterpenoids is well known^64, 65^. Nrf2 is an essential cytoprotective factor that regulates expression of anti-oxidant, anti-inflammatory and detoxifying enzymes which are considered mediators of the cancer chemopreventive activity of CDDO and its derivatives^66–68^. Similarly, EDN-1 is a stress response gene that is highly expressed in DCs and is known to be repressed by CDDO derivatives in an Nrf2 dependent manner^69, 70^. EDN-1 receptors are upregulated on mature DCs and the disruption of autocrine endothelin signaling impairs IL-12 production and T cell stimulation by DCs^18^. In EAE, overexpression of EDN-1 exacerbates disease severity and promotes Th1 and Th17 cell expansion^71^. Lastly, recent data point to significant Nrf-2 dependent mechanisms for the repression of inflammatory cytokines^72^, thus it is conceivable that a tandem regulation of both Nrf2 and NF-κB by CDDO-DFPA and other triterpenoids may work in concert to control the induction of a TolDC phenotype.

Here we show that exposure to CDDO-DFPA *in vivo* during the phase of EAE induction results in an increase in Tregs and suppression of both Th1 and Th17 differentiation. The modified molecular signature of DCs induced by CDOO-DFPA treatment can influence the cytokine environment, which in turn can modulate T cell differentiation either directly or indirectly through modulation of the macrophage phenotype. It is known that during the early phase of EAE induction, microglia/macrophages are rapidly induced to become classically activated M1 cells, release pro-inflammatory cytokines and damage CNS tissue. However, during later phase of EAE, the inflamed CNS contains less activated M2 cells, which release anti-inflammatory cytokines and accompany the resolution of inflammation and concomitant tissue repair. Therefore, the balance between activation and polarization of M1 cells and M2 cells in the CNS is important for disease progression and ultimately resolution. Inhibition of NO production by iNOS inhibitors has been shown to suppress macrophage activation and T cell recruitment into the CNS which ultimately protects mice from neurodegeneration and EAE^73^. Like other CDDO derivatives, CDDO-DFPA is also reported to reduce NO production by macrophages^22^, that may directly suppress macrophage activation. In contrast, the pro-inflammatory cytokines IFN-γ and IL-12 are known to drive M1 cell polarization, while IL-4 drives M2 cell polarization^35^. Since, CDDO-DFPA exposed DCs exhibit decreased IFN-γ and IL-12 and increased IL-4 release, these in turn may have influenced the macrophage population in favor of an M2 phenotype. We also find that CDDO-DFPA-exposed DCs are impaired in their capacity to promote Th17 differentiation *in vitro* and that MOG-pulsed DCs fail to passively induce EAE if pretreated with CDDO-DFPA. Prior reports show the adoptive transfer of immature DCs induces immune tolerance in EAE by suppressing production of both IL-17 and IFN-γ, and by increasing IL-10 secretion^74^. However, there is a notable, and intriguing difference in our study as DCs exposed to CDDO-DFPA also retain characteristics of mature DCs, including the expression of cell surface receptors (e.g., CD80, CD86, MHC-II, PD-L1; Figure S3) and the capacity for antigen uptake (Figure S4). It remains possible that CDDO-DFPA may also modulate these parameters in a manner dependent on when DCs are exposed to CDDO-DFPA relative to DC activation stimuli. However, it is clear that the induction of factors like HO-1, TGF-β, and IL-10 by CDDO-DFPA enable DCs to work through paracrine mechanisms to suppress T cells responses^75^.

The findings reported here certainly have relevance to the role of DCs in MS, but they also hold implications for the potential therapeutic applications of new synthetic triterpenoids in the context of autoimmune and inflammatory disorders. In EAE DCs preferentially accumulate within the spinal cord and cerebellar lesions and cluster with T cells at the peak of EAE^76^. Similarly, DCs accumulate in the CSF and CNS lesions of patients with MS^77^, and circulating DCs increase in the blood of MS patients with an elevated pro-inflammatory cytokine profile^78^. The capacity of triterpenoids to induce an immune suppressive, ‘tolerogenic’ DC phenotype highlights a mechanism that may be exploited in the treatment of multiple sclerosis and other autoimmune disorders, including for the *ex vivo* expansion of autologous DCs with a unique tolerogenic phenotype.

## Materials and Methods

### Animals

Nrf2 knockout, C57BL/6, BALB/c mice, and OT-II T cell receptor (TCR) transgenic mice were purchased from the Jackson Laboratory. All studies were performed in compliance with procedures approved by the Case Western Reserve University School of Medicine’s Institutional Animal Care and Use Committee.

### Reagents and antibodies

CDDO-DFPA was provided by Reata Pharmaceuticals. GM-CSF, IL-4 (Peprotech Inc.), lipopolysaccharides (LPS), β-mercaptoethanol, phorbol 12-myristate 13-acetate (PMA), ionomycin (Sigma Aldrich Inc.), *Mycobacterium Tuberculosis*, incomplete Freund’s adjuvant, pertussis toxin (PTX) (BD bioscience), MOG (35–55) peptide (21stCentury Biochemicals), and EW-7197 (Apexbio Inc.) were purchased from respective manufacturers. Phospho- and total IκBα (Cell Signaling Technology) and NF-κB p65 antibodies (Abcam Inc.) were used for Western blotting. Antibodies for flow cytometry and immunohistochemistry were obtained from either BD bioscience or eBioscience Inc.

### Preparation and characterization of bone marrow-derived dendritic cells

Bone marrow-derived dendritic cells (BMDCs) were expanded from hematopoietic progenitors isolated from C57BL/6 mice^79^. After red cell lysis with ACK buffer, progenitors were cultured in RPMI-1640 plus L-glutamine, 10% FBS, 50 nM β-mercaptoethanol, and 5% penicillin/streptomycin. Fresh medium with 15 ng/ml of GM-CSF and 10 ng/ml of IL-4 were added to cultures on day 0, 3, and 5. BMDCs were harvested on day 7 and analyzed by flow cytometry for CD11c expression (70–80% of the expanded cell population).

### Induction and evaluation of EAE

EAE was induced in 8–10 week old female C57BL/6 mice as previously described^32^. Mice received either 1 mg/kg of CDDO-DFPA by i.p. injection daily, from day 3 to day 15, or vehicle control. Methods for passive induction of EAE by *ex vivo* expanded BMDCs were adopted from previously described protocol^80^. In brief, mice received 2 × 10^6^ LPS-stimulated, MOG-pulsed CD11c+ BMDCs at a total volume of 200 μL injected subcutaneously (100 μL into each hind leg). On the day of BMDC transfer and 48 hours later, each mouse also received 200 ng of pertussis toxin, and DCs were injected into C57BL/6 mice once each week for four consecutive weeks. Mice were monitored daily for survival and symptoms of EAE, using standard criteria^32^.

### Cell processing, fractionation and Western Blotting

Preparation of total cellular lysates and nuclear and cytoplasmic fractions for Western blotting was performed as previously described^81, 82^. Nuclear-cytoplasmic fractions were separated using NE-PER nuclear and cytoplasmic extraction reagents (Thermo Fischer Scientific Inc.), containing a protease and phosphatase inhibitor (Roche Inc.).

### Histology and immunohistochemistry

Spinal cord sections were prepared and stained to study pathology and immune infiltration and BMDCs were stained for immunocytochemical localization as described earlier^82^. Images were acquired using a confocal microscope and combined using LSM 510 imaging software (Carl Zeiss, Inc.).

### Cytokine array and qRT-PCR

Total RNA was isolated using RNAqueous®-Micro Total RNA Isolation Kit (ThermoFisher Scientific Inc.) and subjected to cDNA synthesis using SuperScript® III CellsDirect™ cDNA Synthesis Kit (ThermoFisher Scientific Inc.). The TaqMan® Array Mouse Immune Response plate (ThermoFisher Scientific Inc.) CFX96 Touch™ Real-Time PCR Detection System (Bio-Rad Inc.) was utilized for immune array analysis according to the manufacturer’s protocol. qRT-PCR of individual genes was performed using the following primers and probes (Applied Biosystems Inc.): TNF-α (Mm00443258), IL-6 (Mm00446190), IL-12a (Mm00434165), IL-23a (Mm01160011). The results were generated by 7900HT Sequence Detection System (Applied Biosystems Inc.) and normalized to GAPDH (Mm99999915).

### *In vitro* T-cell allogeneic and syngeneic proliferation assays

For the *in vitro* allostimulation model, splenic T cells were isolated from C57BL/6 mice using the Pan T Cell Isolation Kit (Miltenyi Biotec Inc.) and labeled with 1 μM of carboxyfluorescein succinimidyl ester (CFSE, BioLegend Inc.) for 15 mins, 37 °C. Splenic DCs were isolated from BALB/c mice using a Pan Dendritic Cell Isolation Kit (Miltenyi Biotec Inc.) and incubated for 1 hour in the absence or presence of CDDO-DFPA (100–400 nM). Subsequently, DCs and T cells were co-cultured in 1:10 ratio. After 6 days, CFSE intensity was measured by flow cytometry. For the syngeneic stimulation model, splenic CD4+ T cells were isolated from OT-II TCR transgenic mice using a CD4+ T Cell Isolation Kit (Miltenyi Biotec Inc.) and labeled with CFSE. Splenic DCs were also isolated from C57BL/6 mice and pretreated as above. DCs and T cells were co-cultured at 1:10 ratio with 100 ng/ml of ovalbumin (OVA) peptide 323–329 (InvivoGen Inc.); the TGF-β receptor inhibitor EW-7197 (5 μM) was added where indicated. After 2 days, CFSE intensity was measured by flow cytometry.

### Intracellular cytokine staining

T cells harvested from mice following EAE induction were stimulated with 50 ng/ml of PMA, 1 μg/ml of ionomycin, and 10 mg/ml of GolgiStop (BD bioscience) for 4 hours. Cells were fixed and permeabilized (BD bioscience/eBioscience Inc.) and analyzed by fluorescence-activated cell sorting (FACS).

### *In vit*ro assay of CD4+ T cell differentiation

BMDCs were cultured for 1 hour in the presence or absence of CDDO-DFPA (100–400 nM), washed, then incubated with 100 ng/ml of LPS for 24 hours. BMDC conditioned medium was collected and stored at −80 °C. Splenic CD4+ T cells were isolated from C57BL/6 mice and divided for culture with media conditioned by BMDC as described above. T cells were stimulated with 3 μg/ml of CD3 and 1 μg/ml of CD28 antibodies. For Th17 differentiation assays, 5 ng/ml of TGF-β and 10 ng/ml of IL-6 were added to the culture medium. For Treg differentiation assays, only 5 ng/ml of TGF-β was added. After 2 and 3 days, respectively, cells were stained with antibodies to CD4, IL-17, Foxp3, and CD25 and assessed by flow cytometry for the frequency of Th17, and Treg subsets.

### Statistical analyses

GraphPad Prism software was used to generate all statistical analyses. Statistical significance was analyzed by either an unpaired Student’s t-test or one-way ANOVA with the Bonferroni corrections. EAE scores were analyzed by performing multiple t-tests with Holm-Sidak analysis, and survival of mice was recorded by Kaplan-Meier survival curve followed by the Mental-Cox log-rank test. Data are expressed as mean ± SD or SEM with α set at 0.05.

## Electronic supplementary material

Dataset 1
