# Supplementary material for: A unique tolerizing dendritic cell phenotype induced by the synthetic triterpenoid CDDO-DFPA (RTA-408) is protective against EAE
Source: Sci Rep. 2017 Aug 29;7:9886. doi: 10.1038/s41598-017-06907-4 (PMC5575165; doi:10.1038/s41598-017-06907-4)
Supplement: Supplementary file 1 — Dataset 1 [file 41598_2017_6907_MOESM1_ESM.pdf]

Supplementary information

A unique tolerizing dendritic cell phenotype induced by the synthetic triterpenoid CDDO-DFPA (RTA-408) is protective against EAE

Hsi-Ju Wei, Tej K. Pareek, Qi Liu, John J. Letterio

Figure S1

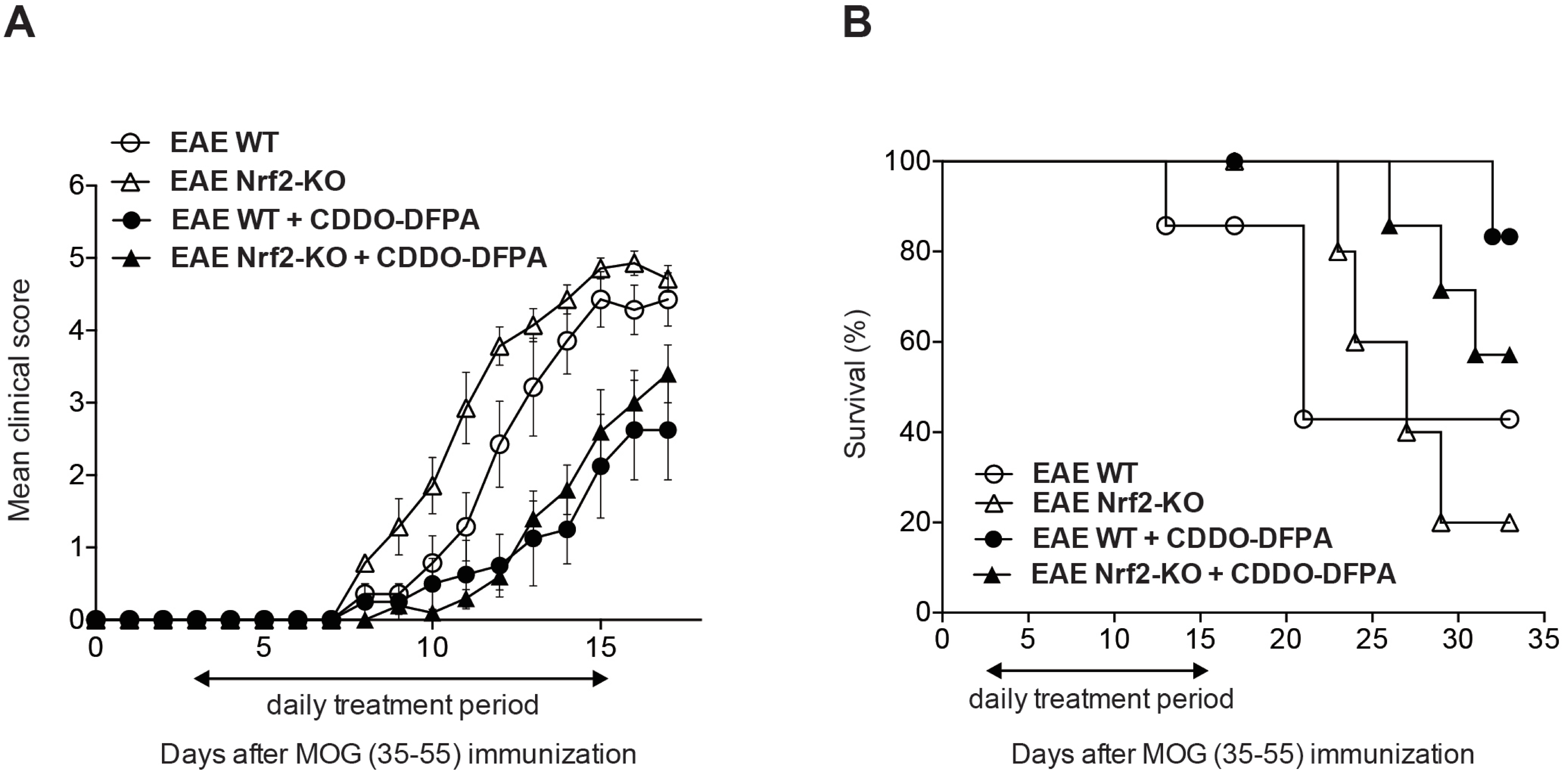

Figure S1. CDDO-DFPA is protective against EAE through Nrf2 independent mechanism. EAE was induced in age-matched female WT or Nrf2-KO C57BL/6 mice (8 to 10 weeks old), by MOG (35-55) immunization. PTX was also injected immediately and again 2 days later. CDDO-DFPA was administered (i.p. injection) daily from day 3 to day 15. (A) A clinical score was assigned to each mouse daily. All data are presented as the mean ± S.E.M. $^*P < 0.05$. Multiple t-tests with Holm-Sidak analysis. (B) Survival curve for immunized mice (Kaplan-Meier survival curve followed by the Mental-Cox log-rank test within 35 days (n= 5-7 mice in each group). $^*P < 0.05$.

Figure S2

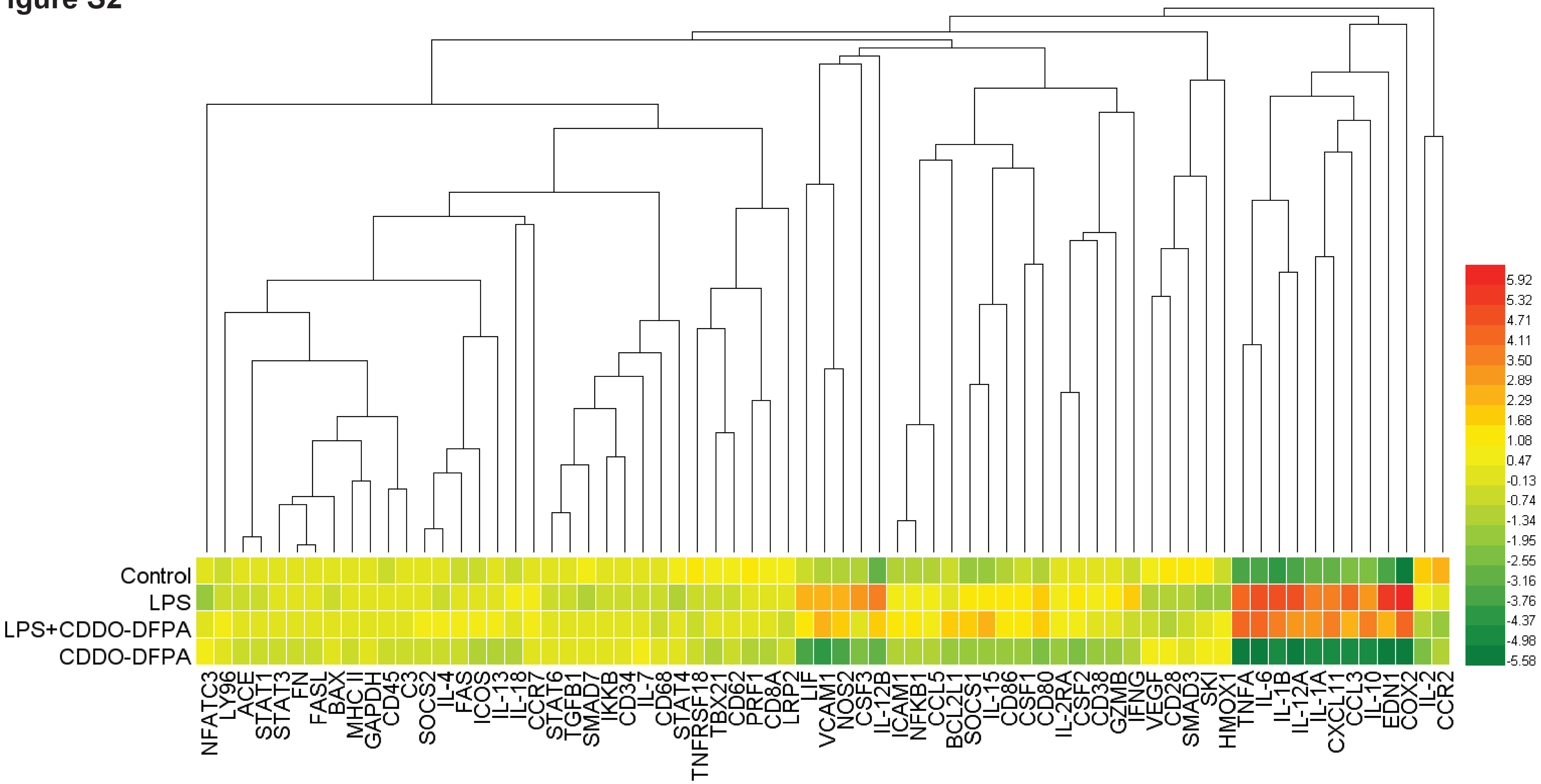

Figure S2. A unique CDDO-DFPA induced transcriptome in LPS-activated BMDCs. Cells were pre-treated in the presence or absence of CDDO-DFPA (200 nM) for 1 hour prior to stimulation with LPS for 3 hours. Cells were then harvested and RNA was extracted for qRT-PCR array. 69 genes were differentially expressed in control, LPS, LPS+CDDO-DFPA, and CDDO-DFPA-treated BMDCs. The heat map was drawn using the HemI (Heat map illustrator) with the default value.

Figure S3

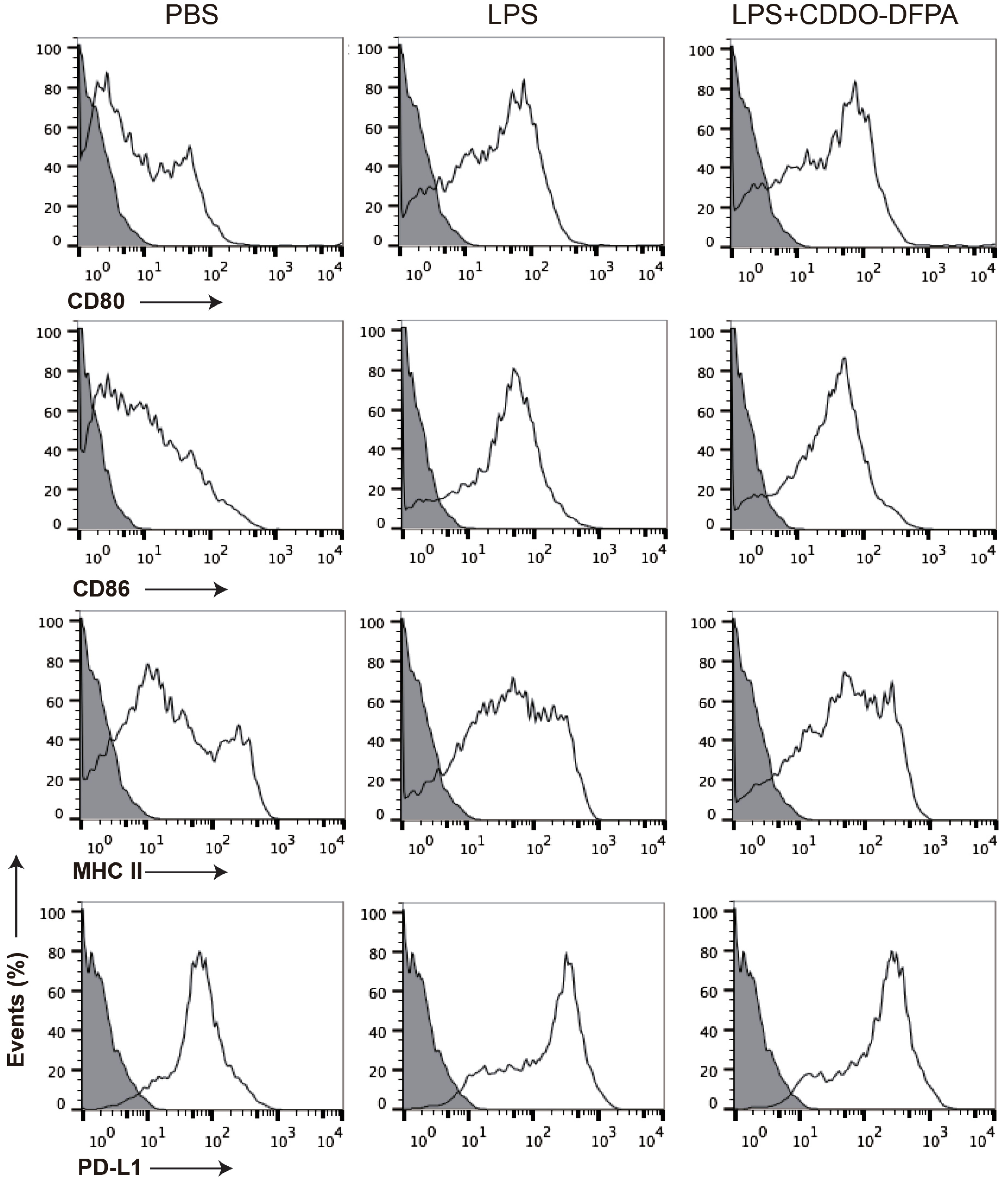

Figure S3. DC cell surface ligand expression is unaltered by CDDO-DFPA. Cells were pre-treated in the presence or absence of CDDO-DFPA (200 nM) for 1 hour prior to stimulation with LPS (100 ng/ml) for 24 hours. Cell surface expression of CD80, CD86, MHC II, and PD-L1 was analyzed following by flow cytometry.

Figure S4

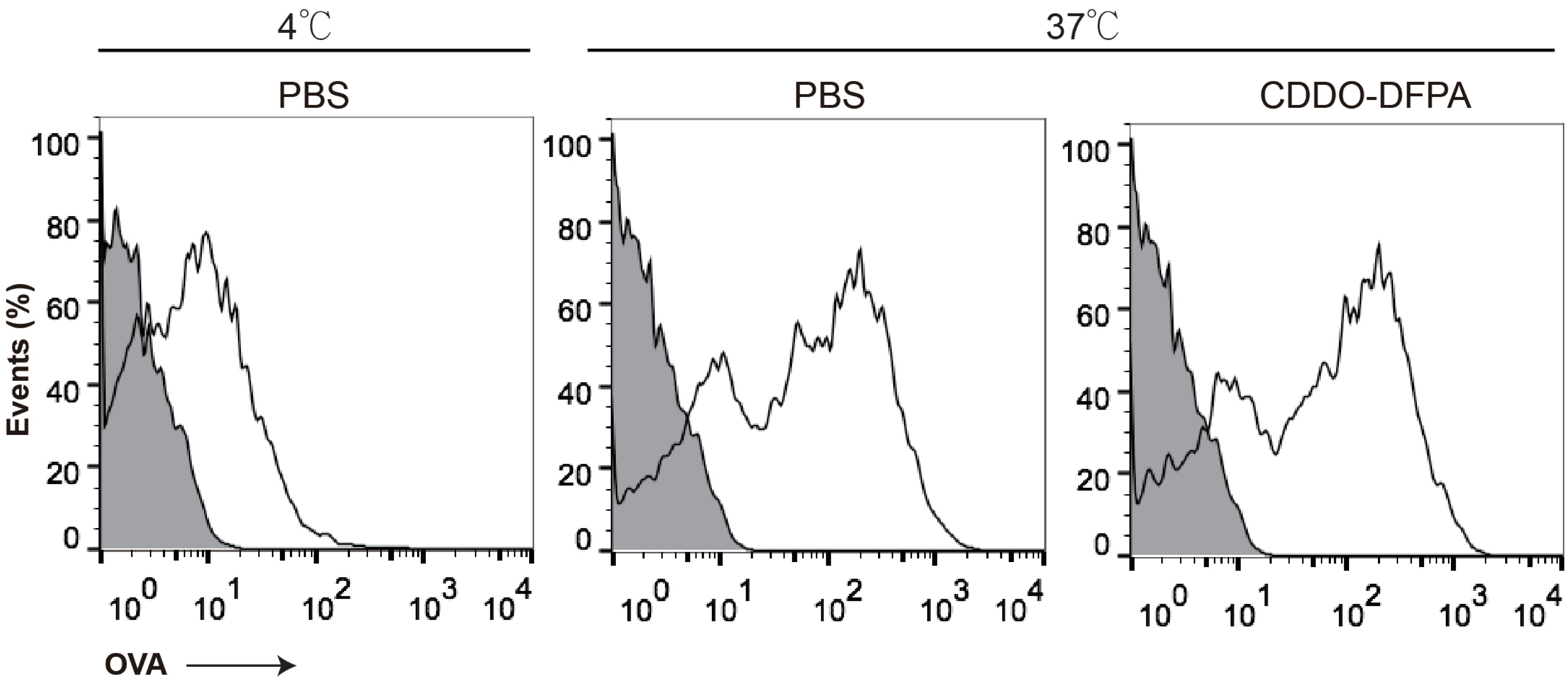

Figure S4. Antigen uptake by BMDCs is not affected by exposure to CDDO-DFPA. Cells were pre-treated in the presence or absence of CDDO-DFPA (200 nM) for 1 hour prior to incubation with OVA-FITC (10 μg/ml) at 4°C or 37°C for another 1 hour. Cells were then washed and analyzed by flow cytometry.

Figure S5

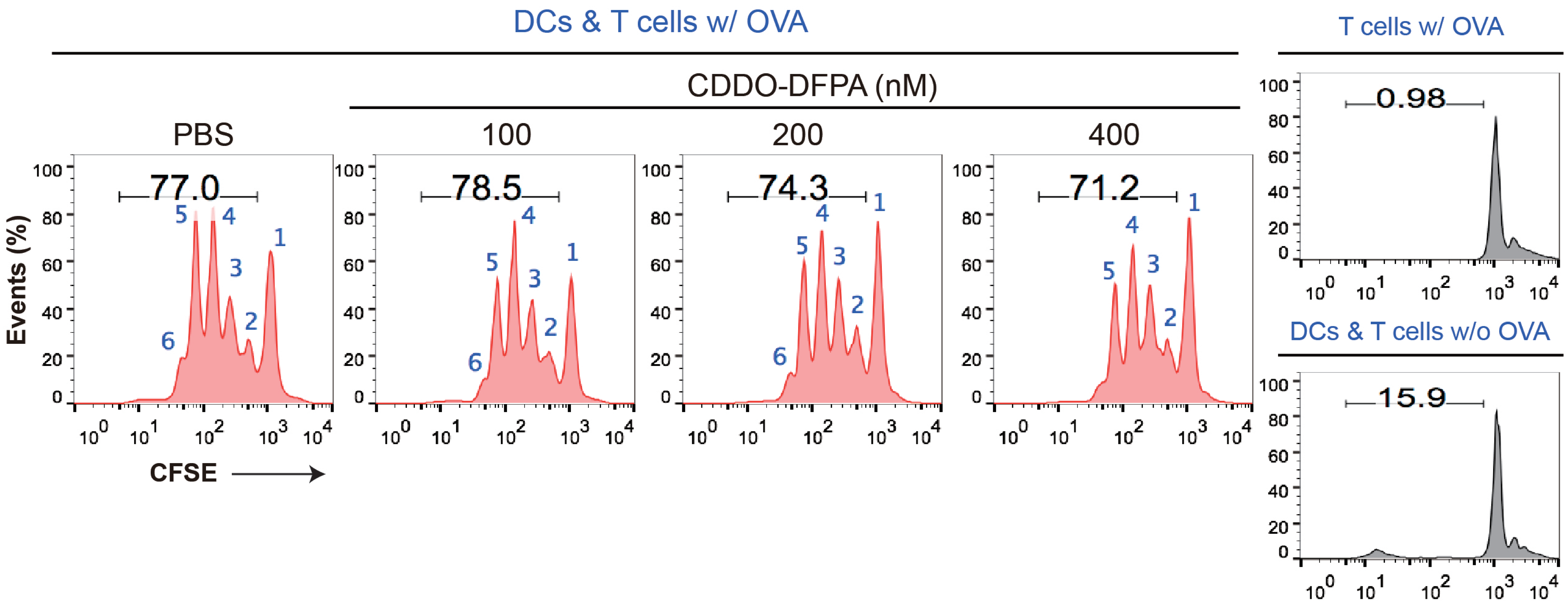

Figure S5. OTII transgenic T cells pretreated with CDDO-DFPA retained responsiveness in DC-OVA-induced T cell proliferation *in vitro*. Splenic T cells and DCs were isolated from OTII transgenic and C57BL/6 mice respectively. T cells were pre-treated with CDDO-DFPA (400 nM) for 1 hour, then washed and stained with CFSE. DCs were co-cultured with T cells at 1:10 ratio with (w/) or without (w/o) OVA incubation. T cell proliferation was determined by flow cytometry at day 2. Numbers indicate the percentage of T cells proliferating and relative to the number of T cell divisions. Similar results were obtained in three independent experiments.

Figure S6

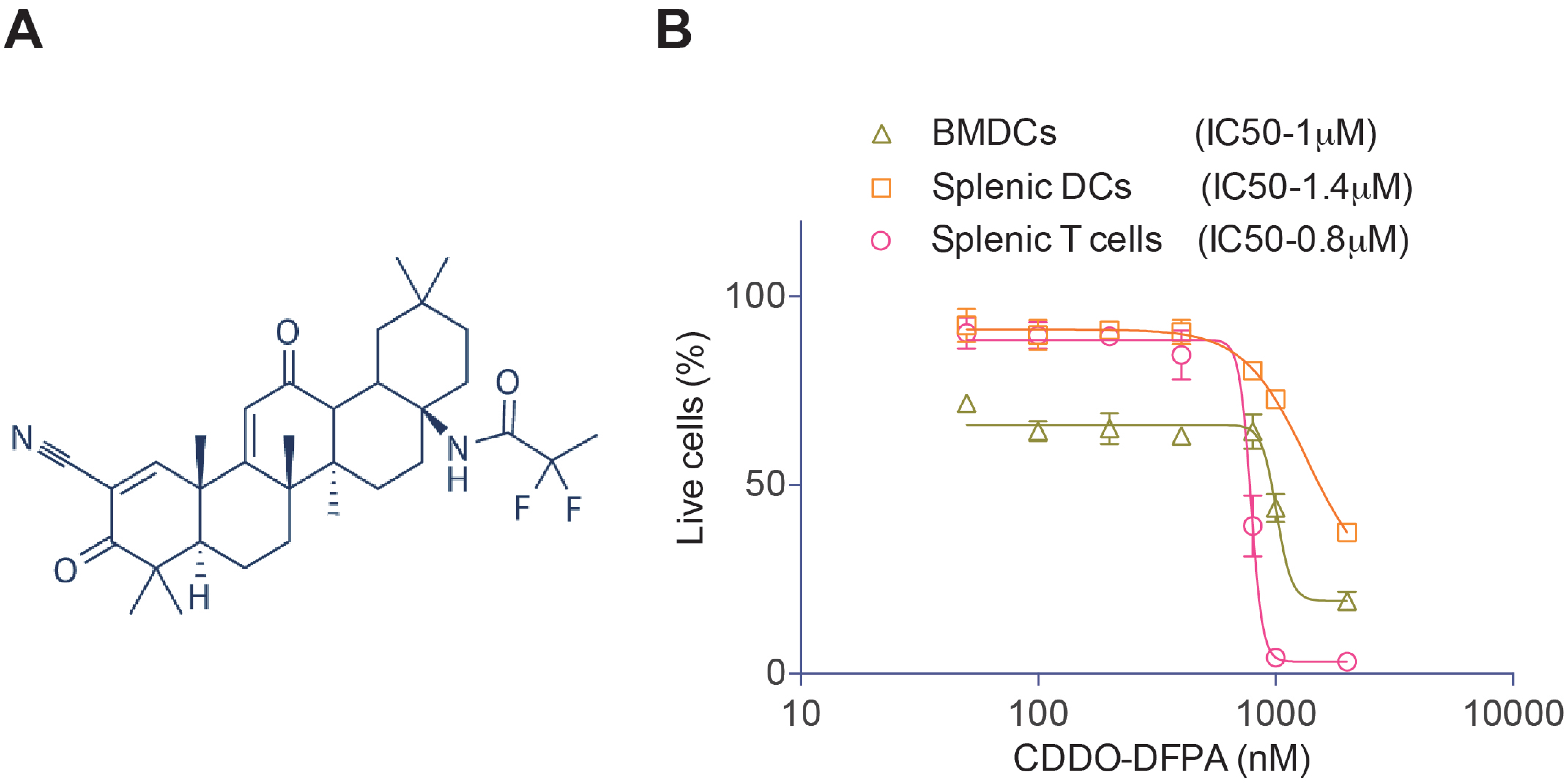

Figure S6. CDDO-DFPA chemical structure and IC50 analysis for in vitro primary cultures of BMDCs, splenic DCs, and T lymphocytes. (A) The chemical structure of CDDO-DFPA. (B) Analysis of the dose-dependent effect of CDDO-DFPA on cell viability in primary cultures assessed at 48 hr. The viability of cells was determined by PI staining. IC50 of CDDO-DFPA was calculated by Graph Pad Prism software.

Figure S7

Figure 2A, phospho-NF-κB p65

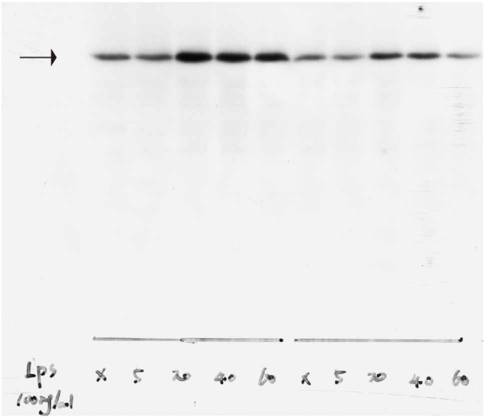

Figure 2A, phospho-NF-κB p65

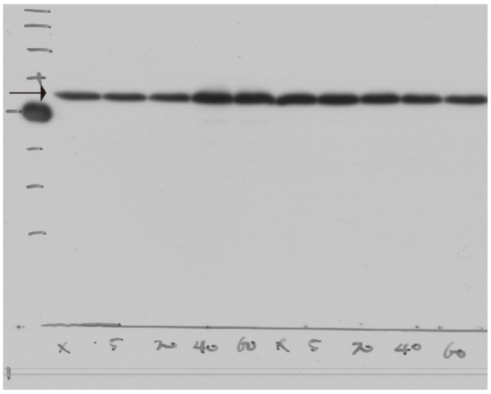

Figure 2A, phospho-IκBα

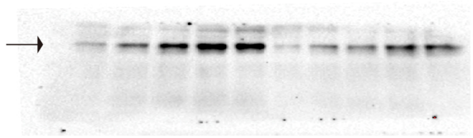

Figure 2A, IκBα

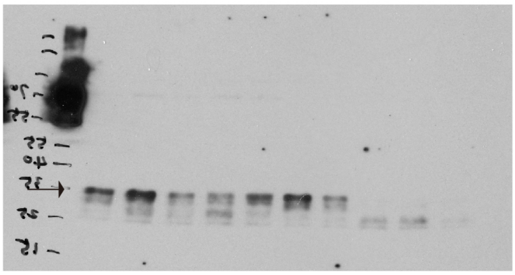

Figure 2A, β-actin

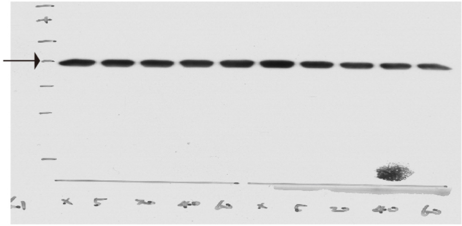

Figure 2B, NF-κB p65

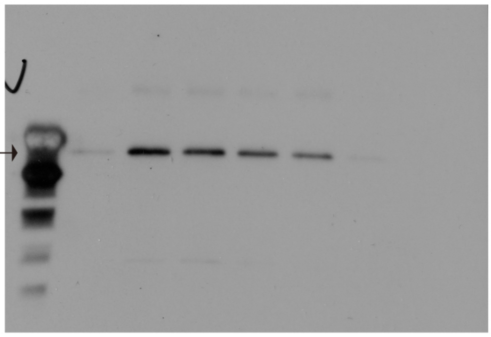

Figure 2B, Lamin B1

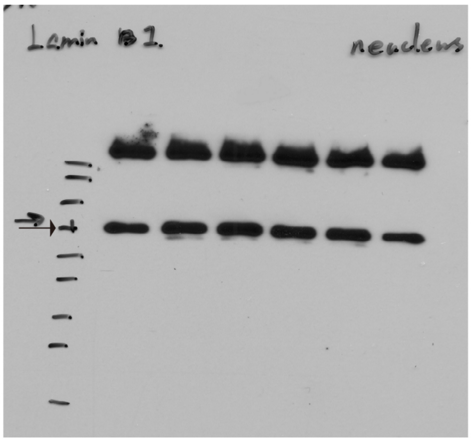

Figure 2B, phospho-IκBα

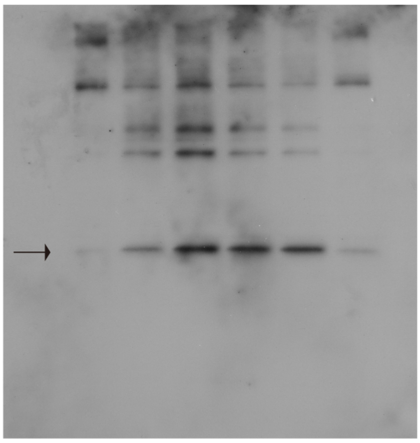

Figure 2B, IκBα

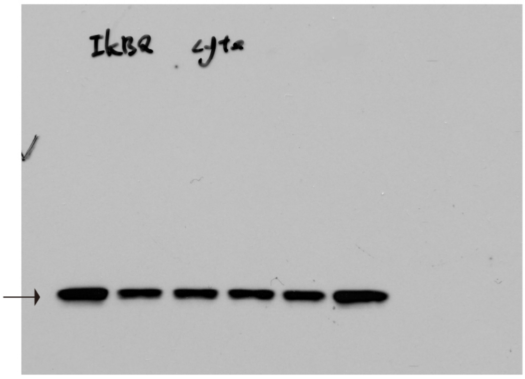

Figure 2B, β-actin

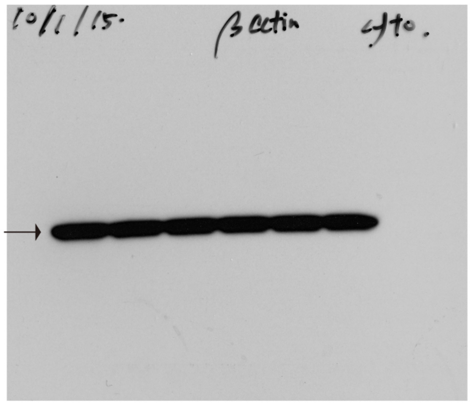

Figure 3G, HO-1

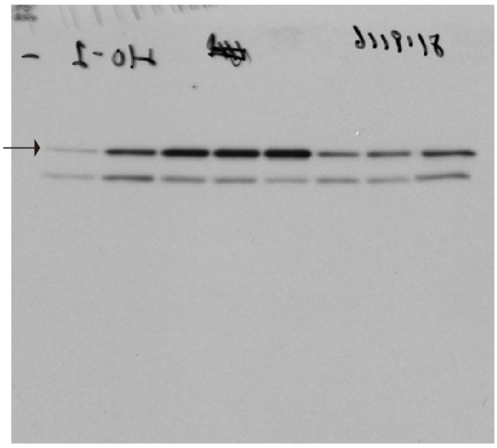

Figure 3G, β-actin

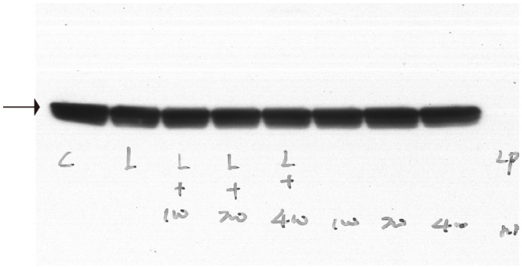

Figure S7. Full scans of Western blotting data
